# Upregulation of FGD6 Predicts Poor Prognosis in Gastric Cancer

**DOI:** 10.3389/fmed.2021.672595

**Published:** 2021-07-05

**Authors:** Jianmin Zeng, Man Li, Huasheng Shi, Jianhui Guo

**Affiliations:** ^1^The Affiliated Hospital of Kunming University of Science and Technology, The First People's Hospital of Yunnan Province, Kunming, China; ^2^The First Affiliated Hospital of Bengbu Medical College, Bengbu, China; ^3^Medical College, Qingdao University, Qingdao, China; ^4^Second Department of General Surgery, The First People's Hospital of Yunnan Province, The Affiliated Hospital of Kunming University of Science and Technology, Kunming, China

**Keywords:** FGD6, gastric cancer, TCGA, prognosis, GSEA

## Abstract

**Background:** The aim of this study was to investigate the prognostic significance of faciogenital dysplasia 6 (FGD6) in gastric cancer (GC).

**Methods:** The data of GC patients from The Cancer Genome Atlas (TCGA) database were used for the primary study. Then, our data were validated by the GEO database and RuiJin cohort. The relationship between the FGD6 level and various clinicopathological features was analyzed by logistic regression and univariate Cox regression. Multivariate Cox regression analysis was used to evaluate whether FGD6 was an independent prognostic factor for survival of patients with GC. The relationship between FGD6 and overall survival time was explored by the Kaplan–Meier method. In addition, gene set enrichment analysis (GSEA) was performed to investigate the possible biological processes of FGD6.

**Results:** The FGD6 level was significantly overexpressed in GC tissues, compared with adjacent normal tissues. The high expression of FGD6 was related to a high histological grade, stage, and T classification and poor prognosis of GC. Multivariate Cox regression analysis showed that FGD6 was an independent prognostic factor for survival of patients with GC. GSEA identified that the high expression of FGD6 was mainly enriched in regulation of actin cytoskeleton.

**Conclusion:** FGD6 may be a prognostic biomarker for predicting the outcome of patients with GC.

## Introduction

Gastric cancer (GC) is the fifth incidence cancer, and it has become the fourth leading cause of cancer deaths in the world, which led to nearly 769,000 deaths in 2020 (1). The treatment strategies for GC include surgery, chemotherapy, radiotherapy, and molecular targeted therapy. Nevertheless, the prognosis remains poor, and the age-standardized 5-year net survival rate was only 20–40% (2), partially due to the majority of GC cases lacking typical symptoms and being diagnosed at an advanced stage with an experience of postsurgical disease relapse or metastasis (3). To this end, new methods of diagnosis or novel molecular biomarkers needed to be discovered for prognosis and reliable therapeutic targets of GC patients.

The modular architectures of faciogenital dysplasia (FGD) family proteins, encompassing FYVE (domain present in Fab1p, YOTB, Vac1p, and EEA1) domains, Dbl homology (DH), and two plectron homology (PH) domains, recognize some phospholipids and proteins such as phosphoinositide (4, 5), phosphatidylinositol 3-phosphate (6), and GTPases, to promote cellular development (7), whose act serves as Rho guanine nucleotide exchange factors (GEFs) (8). There are seven members of the FGD family, namely, FGD1, FGD2, FGD3, FGD4, FGD5, FGD6, and FRG (9). For example, several researches have revealed that FGD family proteins regulated culler functions by specifically activating Cdc42 (10–12).

FGD6, also known as ZFYVE24, is localized on the *Homo sapiens* chromosome 12q22 and consists of 1,400 amino acids. Previous studies have reported that variations in FGD6 increase individual susceptibility to polypoidal choroidal vasculopathy (13). Furthermore, it was also demonstrated that FGD6 coordinates cell polarity and endosomal membrane recycling in osteoclasts by regulating the assembly of different actin-based protein networks and activating Cdc42 at different locations (14). However, to date, little is investigated about the relationship between the expression level of FGD6 and the prognosis of GC.

In the present study, by using bioinformatics analysis in TCGA database, we identified that FGD6 was increased in GC tissues compared with adjacent non-cancerous tissues, which was also verified in paired GC tissues by using GSE63089. Herein, we found that GC patients with higher FGD6 in the tumor tissues had shorter overall survival and poorer clinical phenotypes. In addition, the gene set enrichment analysis (GSEA) was used to investigate the signaling pathways of FGD6 in patients with GC. All data presented that FGD6 may serve as a promising prognostic marker for GC.

## Materials and Methods

### The Extraction Process of the GC Patients' Data

The RNA sequencing data with 373 cases, which include 343 GC patients and 30 adjacent normal tissues, and the clinical information with 406 GC patients were downloaded from the TCGA Genomic Data Commons data portal (https://portal.gdc.cancer.gov/repository). The different expressions of FGD6 were analyzed by the R package limma function, and its prognosis-related values were analyzed by survival analysis. The expression data of GSE63089 with no clinical data in the National Center for Biotechnology Information (NCBI) Gene Expression Omnibus (GEO) database (https://www.ncbi.nlm.nih.gov/geo/) were analyzed to validate the FGD6 expression level by the R package limma function. The details included survival status, age, gender, histological grade, stage, T classification, N classification, and M classification (Table 1). The GSE15459 dataset was used to analyze the clinicopathological factors and the relationship between FGD6 and overall survival time. The clinicopathological data included sex, age, stage, and survival status (Table 2). Patients who were followed up for <30 days and unknown or incomplete clinical information were excluded from TCGA database.

**Table 1:** Characteristics of patients with GC from TCGA.

**Clinical characteristics**		**Total**	**%**
TCGA		406	100
Survival status	Survival	265	65.27
	Death	141	34.73
Age	<65 years	171	42.12
	≥65 years	232	57.14
	Unknown	3	0.74
Gender	Male	150	36.95
	Female	256	63.05
Histological grade	G1	10	2.46
	G2	149	36.70
	G3	240	59.11
	GX	7	1.72
Stage	I	56	13.79
	II	118	29.06
	III	167	41.13
	IV	39	9.61
	Unknown	26	6.40
T classification	T1	23	5.67
	T2	85	20.94
	T3	185	45.57
	T4	103	25.37
	TX	10	2.46
M classification	M0	361	88.92
	M1	27	6.65
	MX	18	4.43
N classification	N0	122	30.05
	N1	109	26.85
	N2	80	19.70
	N3	78	19.21
	NX	15	3.69
	Unknown	2	0.49

**Table 2:** Clinical characteristics of patients with GC from GSE15459.

**Clinical characteristics**		**Total**	**%**
GEO		192	100
Survival status	Survival	97	50.52
	Death	95	49.48
Age	≤66 years	92	47.92
	>66 years	100	52.08
Gender	Male	125	65.10
	Female	67	34.90
Stage	I	31	16.15
	II	29	15.10
	III	72	37.50
	IV	60	31.25

### Clinical Specimens

Twenty cases of GC tissues and normal tissues were collected from RuiJin Hospital, Shanghai Jiaotong University, and have been approved by the Ethics Committee of RuiJin Hospital, Shanghai Jiaotong University. Tissues were preserved in a −80°C refrigerator after surgical excision. All patients had given written informed consent before the study. None of the patients had received preoperative radiotherapy or chemotherapy before surgical resection.

### Quantitative Real-Time Polymerase Chain Reaction Analysis

The total RNA was extracted from GC tissues and normal tissues by a TRIzol reagent (Invitrogen, USA), following the manufacturer's instructions. Reverse transcription was performed to obtain cDNA by 5^*^fastking, and qRT-PCR was performed with the SYBR Green Master Mix (Thermo Fisher Scientific, USA). Sequences were as follows: for FGD6, forward 5′-CAGCCTGGTCGGGTTTTTCT-3′ and reverse 5′-CAGCCAGTGAGAGCATGTTG-3′; for GAPDH, forward 5′-GACTCATGACCACAGTCCATGC-3′ and reverse 5′-AGAGGCAGGGATGATGTTCTG-3′. The relative mRNA expression level was calculated by the 2^−Δ*CT*^ method.

### GSEA

GSEA 4.1.0 was used to detect the potential mechanism of FGD6 and showed statistically significant differential expression between high and low expression groups based on the median expression of FGD6. GESA was conducted according to the default weighted enrichment statistics for 1,000 times. Gene sets with normalized (NOM) *p*-value < 0.05 and false discovery rate (FDR) < 0.25 were considered significantly enriched.

### Statistical Analysis

All statistical analyses in our study were carried out using R software (V.3.5.3) and IBM SPSS statistical software (version 22.0). The differences between groups were analyzed using the Wilcoxon test and logistic regression. Clinicopathological characteristics related to overall survival in GC patients were verified using Cox regression and the Kaplan–Meier method. Multivariate Cox analysis was used to evaluate the relationship between the clinicopathological features and FGD6 expression. The cutoff value for FGD6 expression was determined based on the median values. The scatter plot of qRT-PCR results in this study was performed using GraphPad Prism 8 software. A *p*-value < 0.05 was considered statistically significant.

## Results

### Overexpression of FGD6 in GC Tissues

**Figure 1:**
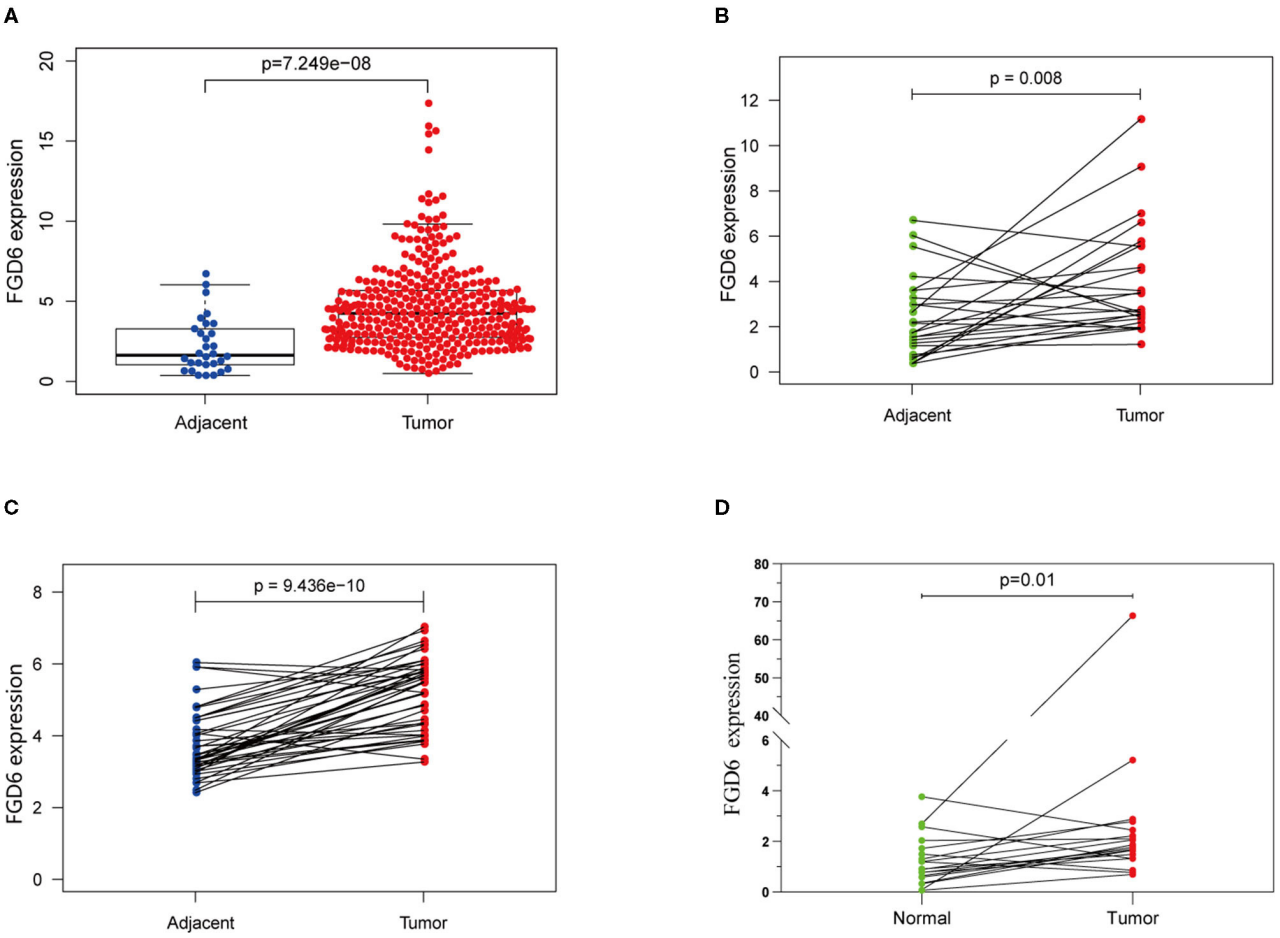
The expression of FGD6 in GC tissues and adjacent normal tissues. **(A)** FGD6 expression is upregulated in TCGA cohort; **(B)** FGD6 is overexpressed in GC tissue compared to para-cancerous tissues in TCGA cohort; **(C)** FGD6 expression is elevated in GC tissues in the GSE63089 dataset; **(D)** FGD6 expression was significantly upregulated in GC compared to normal tissues by qRT-PCR.

Results of the data from TCGA database, as shown in Figure 1A, revealed that the mRNA expression of FGD6 was significantly upregulated in GC tissues compared with adjacent normal stomach tissues (*p* = 7.249e^−8^). In addition, the mRNA expression of FGD6 was compared in 30 pairs of tumor and adjacent normal tissues, revealing its expression enhanced in tumor tissues compared with the adjacent normal tissues (*p* = 0.008) (Figure 1B). Similarly, the result of FGD6 mRNA expression also showed the same phenomenon by using the GSE63089 dataset (*p* = 9.436e^−10^) (Figure 1C). Consistently, qRT-PCR analysis showed that FGD6 expression at the mRNA level was higher in GC tissues relative to that in corresponding non-cancerous tissues (*P* = 0.01) (Figure 1D).

### Correlation Between Expression of FGD6 and Clinical Features of GC Patients

**Figure 2:**
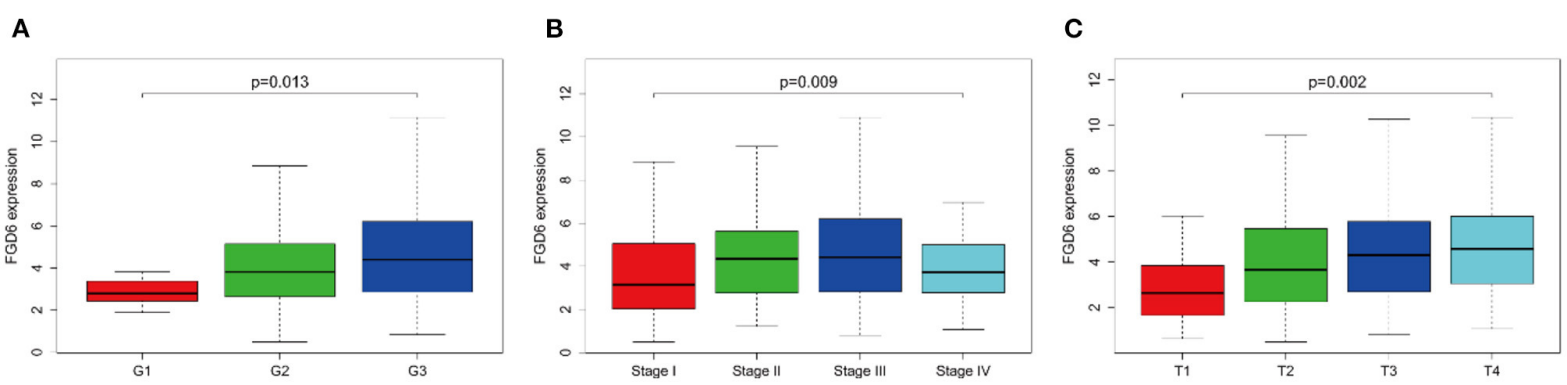
The link between FGD6 mRNA expression and clinicopathologic features in GC patients. **(A)** Histological grade, **(B)** Clinical stage, and **(C)** T classification.

As shown in Figure 2, the clinical parameters of GC patients from TCGA were analyzed, showing that the mRNA expression of FGD6 was related to histological grade (*p* = 0.013), stage (*p* = 0.009), and T classification (*p* = 0.002). Logistic regression analysis revealed that overexpression of FGD6 in GC was significantly correlated with high histological grade (OR = 8.385 for G3 vs. G1, *p* = 0.049), stage (OR = 2.730 for II vs. I, *p* = 0.006; OR = 2.667 for III vs. I, *p* = 0.006; OR = 2.625 for IV vs. I, *p* = 0.037), and high T classification (OR = 4.293 for T2 vs. T1, *p* = 0.030; OR = 5.684 for T3 vs. T1, *p* = 0.007; OR = 7.619 for T4 vs. T1, *p* = 0.002) (Table 3). These findings indicated that GC patients with high FGD6 expression have more chances to develop an advanced GC than GC patients with low FGD6 expression.

**Table 3:** FGD6 expression associated with clinical pathological characteristics (logistic regression).

**Clinical characteristics**	**Total**	**FGD6 OR (95% CI)**	***P*-value**
Age(≥65 vs. <65 years)	341	0.966 (0.628–1.486)	0.876
Sex (male vs. female)	343	0.791 (0.509–1.226)	0.295
**Grade**			
G2 vs. G1	136	5.800 (0.992–110.485)	0.105
G3 vs. G1	328	8.385 (1.455–158.226)	0.049
**Stage**			
II vs. I	152	2.730 (1.350–5.728)	0.006
III vs. I	185	2.667 (1.354–5.455)	0.006
IV vs. I	84	2.625 (1.071–6.606)	0.037
**T classification**			
T2 vs. T1	93	4.293 (1.294–19.585)	0.030
T3 vs. T1	176	5.684 (1.086–25.133)	0.007
T4 vs. T1	104	7.619 (2.324–34.552)	0.002
**M classification**			
M1 vs. M0	327	1.618 (0.689–3.991)	0.277
**N classification**			
(N1 + N2 + N3) vs. N0	327	1.480 (0.925–2.3799)	0.103

### High Expression of FGD6 Predicts Poor Overall Survival in GC

**Figure 3:**
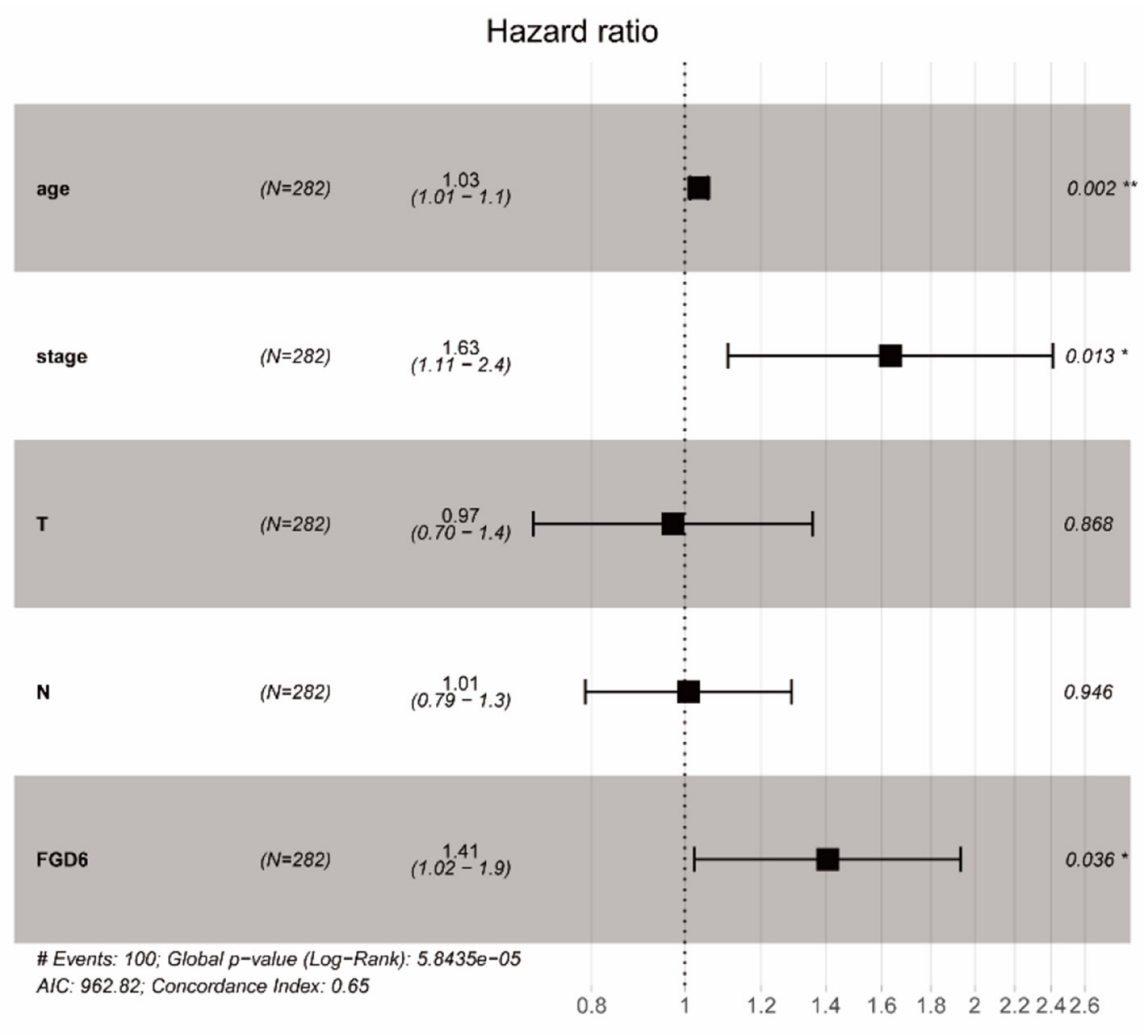
Forest plot of the correlation between FGD6 expression and GC patient overall survival. *P < 0.05, **P < 0.01.

**Figure 4:**
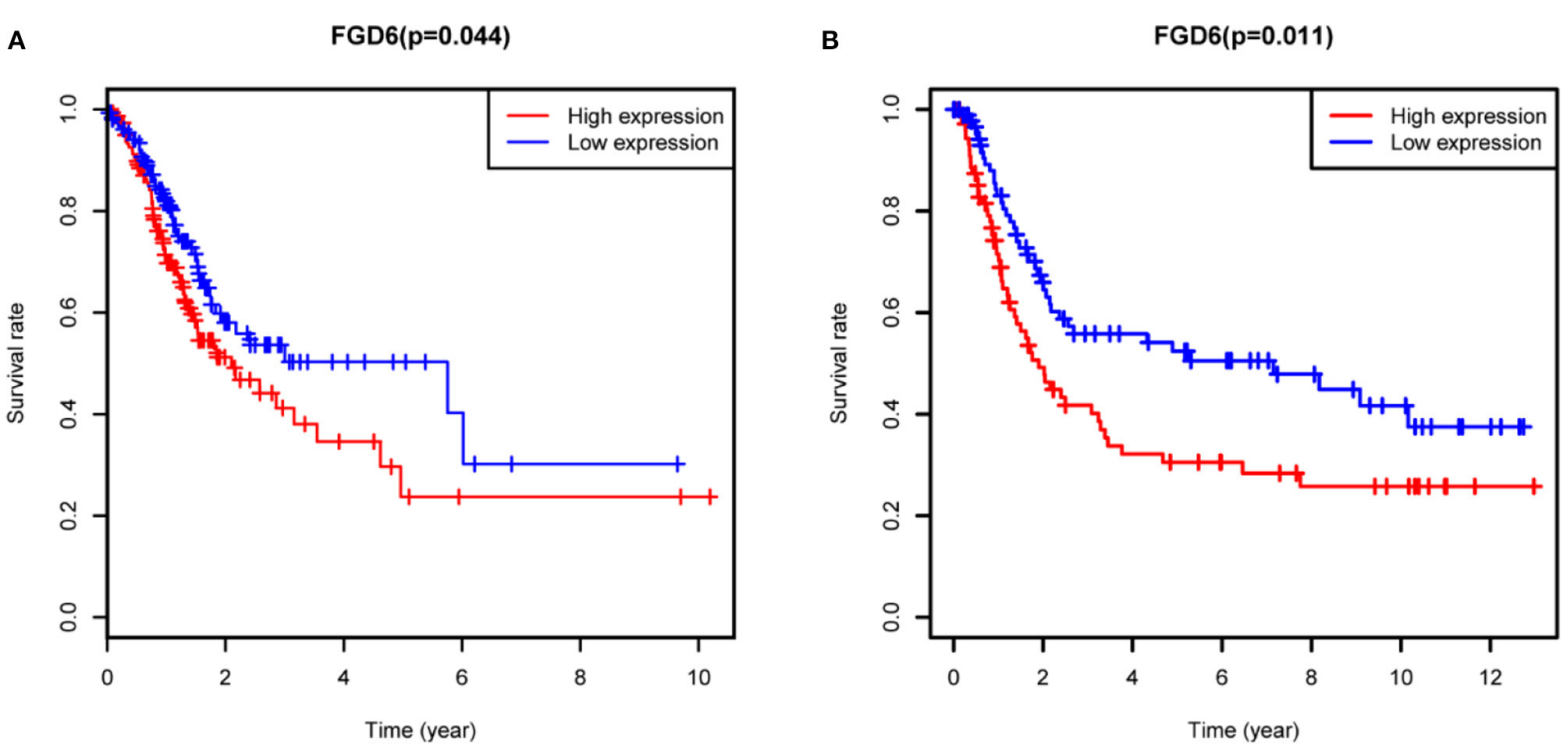
Kaplan-Meier analysis for overall survival of GC patients with FGD6 expression. **(A)** TCGA cohort. **(B)** GSE15459 dataset.

To evaluate the diagnostic value of FGD6, univariate and multivariate Cox regressions were used to analyze the impact of FGD6 expression and other clinicopathological factors on survival (Table 4). In univariate analysis, age (HR = 1.026, *p* = 0.009), stage (HR = 1.483, *p* = 0.001), N classification (HR = 1.244, *p* = 0.018), and FGD6 (HR = 1.440, *p* = 0.022) were related to overall survival in patients with GC. In multivariate analysis, FGD6 expression (HR = 1.406, *p* = 0.036) was significantly associated with poor overall survival, similar to age (HR = 1.034, *p* = 0.002) and stage (HR = 1.634, *p* = 0.013) (Figure 3). Furthermore, univariate Cox regression analysis showed that stage (HR = 2.789, *p* = 3.150e^−14^) and FGD6 expression (HR = 1.567, *p* = 0.001) were associated with poorer survival in the GSE15459 dataset. Multivariate Cox regression analysis of the above factors found that FGD6 expression (HR = 1.374, *p* = 0.027) and stage (HR = 2.764, *p* = 1.500e^−13^) were independent prognostic factors of GC in the GSE15459 dataset (Table 5). Kaplan–Meier survival curves showed that patients with high expression of FGD6 exhibited poorer survival time compared with those with low expression of FGD6 (Figure 4A, *p* = 0.044). In the same way, this result was further validated in the GSE15459 dataset (Figure 4B, *p* = 0.011).

**Table 4:** Univariate and multivariate Cox regression analyses of the correlation of prognostic factors with overall survival among GC patients by TCGA database.

**Clinicopathologic**	**Univariate analysis**		**Multivariate analysis**	
**variable**				
	**HR (95% CI)**	***P*-value**	**HR (95% CI)**	***P*-value**
Age	1.026 (1.006–1.046)	0.009	1.034 (1.013–1.056)	0.002
Sex	1.528 (0.992–2.354)	0.054		
Histological grade	1.221 (0.829–1.798)	0.311		
Stage	1.483 (1.171–1.878)	0.001	1.634 (1.108–2.409)	0.013
T classification	1.281 (1.000–1.642)	0.050	0.972 (0.696–1.358)	0.868
M classification	1.891 (0.951–3.763)	0.069		
N classification	1.244 (1.039–1.490)	0.018	1.008 (0.788–1.291)	0.946
FGD6	1.440 (1.053–1.971)	0.022	1.406 (1.023–1.933)	0.036

**Table 5:** Univariate and multivariate Cox regression analyses of the correlation of prognostic factors with overall survival among GC patients by the GSE15459 dataset.

**Clinicopathologic**	**Univariate analysis**		**Multivariate analysis**	
**variable**				
	**HR (95% CI)**	***P*-value**	**HR (95% CI)**	***P*-value**
Age	1.000 (0.984–1.016)	0.970		
Sex	1.402 (0.908–2.165)	0.127		
Stage	2.789 (2.140–3.635)	3.150e^−14^	2.764 (2.111–3.620)	1.500e^−13^
FGD6	1.567 (1.190–2.063)	0.001	1.374 (1.036–1.821)	0.027

### Signaling Pathways Associated With FGD6 Expression

**Figure 5:**
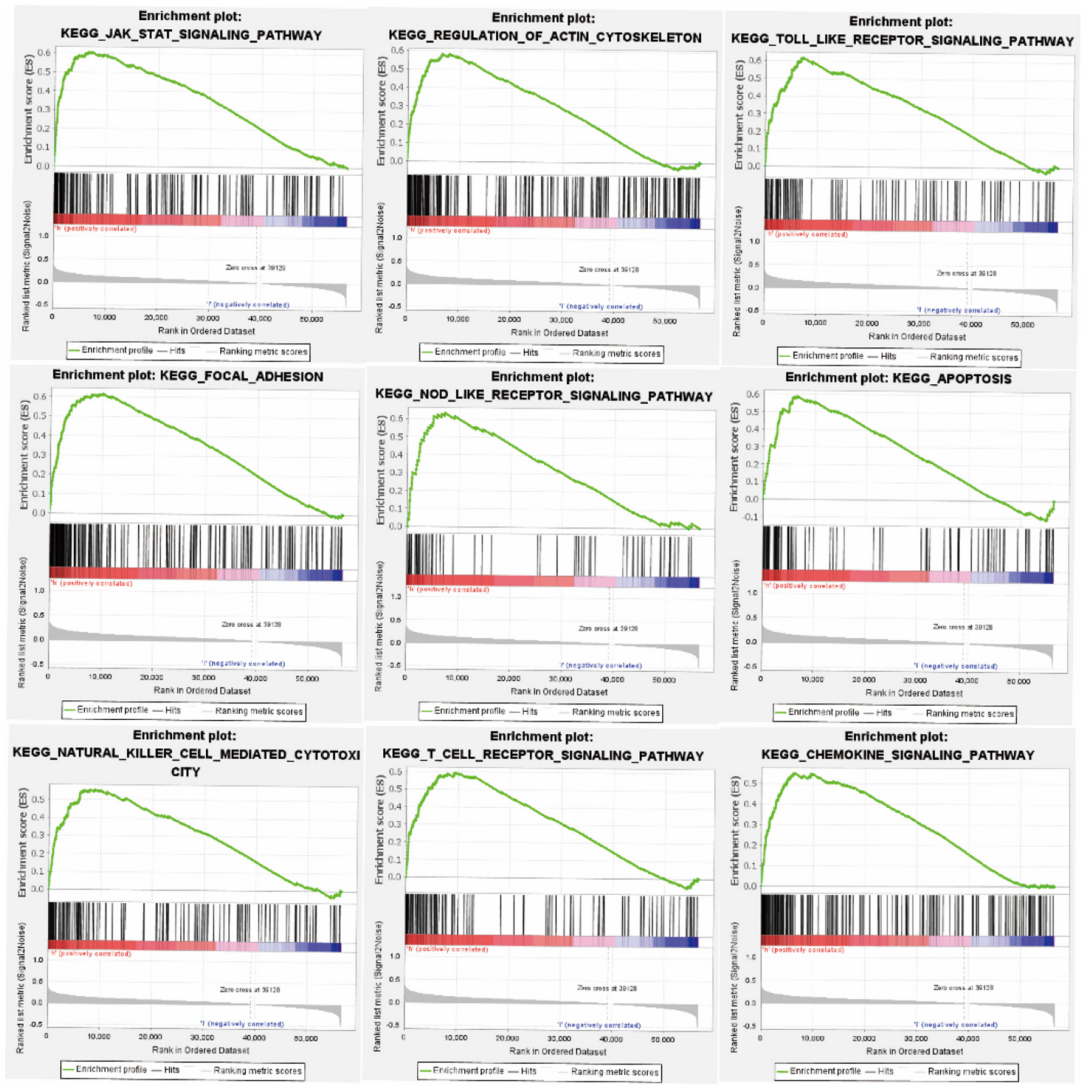
GSEA enrichment plots. The GSEA results suggested that the significantly enriched signaling pathways were the JAK/STAT signaling pathway, regulation of actin cytoskeleton, Toll-like receptor signaling pathway, focal adhesion, apoptosis, NOD-like receptor signaling pathway, natural killer cell-mediated cytotoxicity, T-cell receptor signaling pathway, and chemokine signaling pathway.

In order to investigate the potential signaling pathways and promising biological function of FGD6 in GC, we explored high and low FGD6 expression datasets though GSEA based on the median expression of FGD6. As shown in Figure 5, GSEA mainly regulated the JAK/STAT signaling pathway, regulation of actin cytoskeleton, Toll-like receptor signaling pathway, focal adhesion, apoptosis, NOD-like receptor signaling pathway, natural killer cell-mediated cytotoxicity, T-cell receptor signaling pathway, and chemokine signaling pathway, which were obviously enriched in the FGD6 high-expression phenotype.

## Discussion

Prognostic biomarkers play an important role in the prognosis of GC, and there has been a lot of literature on the biomarkers of GC (15). However, the current conventional prediction models for GC rely on clinical prognostic factors such as age, TNM stage, and pathological grade. Prognostic assessment of patients with GC remains a challenging issue. A non-invasive or less-invasive test with tumor-specific biomarkers to facilitate diagnosis, recurrence assessment, and personalized chemotherapy for GC patients is urgently required.

GEFs are associated with numerous cellular responses such as proliferation, differentiation, and motility though activating Rho-family GTPases (16). Rho-family GTPases are activated by GEFs, which leads to aberrant signaling from growth factor receptors such as transmembrane receptor tyrosine kinases and G protein-coupled receptors in cancer (17). The FGD family contains the DH and PH1 domains that can reconcile activation of Rho GTPases by acting as GEFs (7). Rho GTPases function as binary molecular switches converted between an active, GTP-bound state and an inactive, GDP-bound state, where the transformation of GDP to GTP is mediated by GEFs (16), and it also was involved in cancer cell invasion and metastasis (18). CDC42 belongs to a member of the Rho family of small GTPases that have been involved in the regulation of multiple signaling pathways (19), and it plays a pivotal role in epithelial-to-mesenchymal transition, cell-cycle progression, migration, invasion, tumor growth, angiogenesis, and oncogenic transformation (20). Researches have demonstrated that FGD6 can activate CDC42 through its PH domains, binding phosphatidylinositol in the vicinity of ligand-bound integrins (14). It was reported that knockdown of CDC42 inhibited the proliferation, migration, and invasion of GC cells (21). Moreover, studies have found that FARP1 binds to integrin αvβ5 and promotes GC cell migration and invasion through the activation of CDC42 (22). Recently, a study has shown that aberrant expression of CDC42 in colorectal cancer can cause the migration, invasion, and metastasis through activation of the VEGF/NRP1 axis and is closely related to the prognosis of the patients (23).

In this study, bioinformatic analysis was performed in RNA-sequencing data of GC, and established the prognostic relevance of FGD6 mRNA expression level to GC patient clinical outcomes based on TCGA database. We identified and verified that FGD6 was highly expressed in GC tissues relative to para-cancerous tissues, as well as in the GSE63089 dataset of GEO database; the FGD6 expression level was significantly overexpressed in GC tissues as compared to the normal gastric tissues, as verified by qRT-PCR, and its expression level was correlated with clinicopathological parameters and GC patient survival. In addition, GSEA suggested that the main functions of high FGD6 expression were enriched in the JAK/STAT signaling pathway, Regulation of actin cytoskeleton, Toll-like receptor signaling pathway, focal adhesion, apoptosis, NOD-like receptor signaling pathway, natural killer cell-mediated cytotoxicity, T-cell receptor signaling pathway, and chemokine signaling pathway.

However, the regulatory mechanism of FGD6 needs to be further illustrated. The actin cytoskeleton assembles with microtubules and intermediate filaments to form branching networks or bundles that polarize cells, support intercellular junctions, and promote cell adhesion and migration (24). For example, it was reported that neuropilin and tolloid-like 1 modulate the expression of cytoskeletal enhanced migration and invasive potential of epithelial ovarian cancer (25). In parallel, actin cytoskeleton reorganization was also associated with cell migration and motility in human breast cancer (18). Earlier reports had identified that Rho GTPases promote cell adhesion and migration *via* reassembling actin cytoskeletons (26–29). Besides, we speculated that FGD6 may provide us with a new direction in invasion and migration of GC.

Whether other genetic mutations cause uncontrolled proliferation of GC was considered, and FGD6 is coincidentally increased. TCGA mutation data were analyzed by the Perl language. Then, the top 30 mutated genes were selected in the GC samples, and the correlation between FGD6 and these genes was analyzed by using the correlation analysis function of the GEPIA2 (http://gepia2.cancer-pku.cn/). The results are shown in Supplementary Data Sheet 1. In future analyses, the specific molecular mechanism of FGD6 regulation of GC is still unclear, which requires some experiments to be conducted *in vitro* and *in vivo*. In summary, our study revealed that the overexpression of FGD6 was a prognostic biomarker and a potentially viable therapeutic agent of GC patients.

## Data Availability Statement

Publicly available datasets were analyzed in this study. This data can be found here: The Cancer Genome Atlas (https://portal.gdc.cancer.gov/). NCBI Gene Expression Omnibus (GSE63089 and GSE15459).

## Ethics Statement

The studies involving human participants were reviewed and approved by RuiJin hospital, Shanghai Jiaotong University. The patients/participants provided their written informed consent to participate in this study.

## Author Contributions

JZ wrote the manuscript and provided the design idea of this study. ML and HS analyzed the data and supplemented the ideas. JG provided some comments for this paper. All authors read and approved the final manuscript.

## Conflict of Interest

The authors declare that the research was conducted in the absence of any commercial or financial relationships that could be construed as a potential conflict of interest.
