# Supplementary material for: Upregulation of FGD6 Predicts Poor Prognosis in Gastric Cancer
Source: Front Med (Lausanne). 2021 Jul 5;8:672595. doi: 10.3389/fmed.2021.672595 (PMC8288026; doi:10.3389/fmed.2021.672595)
Supplement: Supplementary file 1 [file Table_1.pdf]

Supplementary Data Sheet 1: The correlation between FGD6 and the top 30 mutated genes in gastric cancer.

**Gene**	**Correlation coefficient**	**P-value**
TTN	R = 0.15	p-value = 0.002
TP53	R = 0.12	p-value = 0.015
MUC16	R = 0.2	p-value = 2.4e-05
ARID1A	R = 0.3	p-value = 2.2e-10
LRP1B	R = 0.038	p-value = 0.42
SYNE1	R = 0.28	p-value = 2e-09
FLG	R = -0.032	p-value = 0.51
FAT4	R = 0.25	p-value = 5.2e-08
CSMD3	R = -0.041	p-value = 0.39
PCLO	R = -0.038	p-value = 0.43
DNAH5	R = 0.16	p-value = 0.00057
KMT2D	R = 0.33	p-value = 8.9e-13
FAT3	R = 0.039	p-value = 0.41
HMCN1	R = 0.23	p-value = 1.1e-06
OBSCN	R = 0.19	p-value = 6.4e-05
ZFHX4	R = 0.22	p-value = 3.6e-06
RYR2	R = 0.14	p-value = 0.0041
SPTA1	R = 0.11	p-value = 0.019
PIK3CA	R = 0.4	p-value = 0
CSMD1	R = -0.022	p-value = 0.65
PCDH15	R = 0.092	p-value = 0.053
DMD	R = 0.14	p-value = 0.0031
AHNAK2	R = 0.31	p-value = 1.7e-11
USH2A	R = 0.062	p-value = 0.19
XIRP2	R = 0.031	p-value = 0.52
PLEC	R = 0.38	p-value = 2.2e-16
LAMA1	R = 0.019	p-value = 0.7
CUBN	R = 0.1	p-value = 0.03
KMT2C	R = 0.37	p-value = 4.4e-16
DNAH9	R = 0.0072	p-value = 0.88
